# A Case of Chronic Aspiration Bronchiolitis

**DOI:** 10.7759/cureus.22562

**Published:** 2022-02-24

**Authors:** Luke Buxton, Mufadda Hasan

**Affiliations:** 1 Pulmonary and Critical Care Medicine, Arrowhead Regional Medical Center, Colton, USA

**Keywords:** progressive dyspnea, chronic hypoxemic respiratory failure, exertional dyspnea, chronic cough, chronic aspiration bronchiolitis, chronic lung disease, bronchiolitis, chronic aspiration, diffuse aspiration bronchiolitis

## Abstract

Chronic aspiration bronchiolitis or diffuse aspiration bronchiolitis is a rare disease characterized by chronic inflammation of the bronchioles due to recurrent aspiration of foreign material. To date, there are limited data on chronic aspiration bronchiolitis because it is often present subclinically or remains undiscovered until autopsy. Additionally, time to diagnosis is often prolonged and includes invasive workup prior to definitive diagnosis. Here, we present a case of lung disease attributed to chronic aspiration after a thorough workup resulted in histopathology consistent with a primary diagnosis of aspiration bronchiolitis. Radiographic and pathologic specimens also demonstrated features of usual interstitial pneumonia adding to the complexity of pathology that can be seen with aspiration diseases.

## Introduction

Aspiration of foreign material into the upper airways and lungs causes a diverse spectrum of pathology with varied clinical presentation. The type of pathology encountered is dependent on the quantity and frequency of aspiration, type of foreign material aspirated, and the host's response to inhaled foreign material [1]. In chronic aspiration diseases, occult aspiration is often present well before the onset of symptoms, complicating mitigation of risk factors before clinically prevalent disease occurs. When evaluating a patient with chronic cough, progressive dyspnea, or increasing sputum production, it is important to consider chronic aspiration as an etiology given the prevalence of these symptoms in patients who suffer from chronic aspiration. Risk factors for chronic aspiration include decreased consciousness, compromised airway defenses, dysphagia, gastroesophageal reflux disease (GERD), and recurrent vomiting [1]. Chest radiography including computerized tomography (CT) often shows patterns of injury related to chronic aspiration. Further workup with bronchoscopy and surgical biopsy is warranted when previous workup is nondiagnostic.

Pathologically, chronic aspiration has been shown to cause a myriad of small airway diseases including bronchiectasis, interstitial lung disease, organizing pneumonia, bronchiolitis obliterans, exogenous lipoid pneumonia, and diffuse aspiration bronchiolitis (DAB) [1]. DAB was first described in 1996 and is characterized by chronic inflammation of the bronchioles due to recurrent aspiration [2]. The autopsy study that included 4880 patients found evidence of DAB in 31 (0.64%) patients with an estimated mean age of 81 years [2]. Further small studies and case series suggest a mean age of patients clinically diagnosed with DAB to be 50 and 56.5 years [3,4]. Typical symptoms include dyspnea, cough, and sputum production [4]. It is often associated with a predisposing factor for aspiration such as GERD or underlying neurologic disorders such as dementia [5]. Radiographic findings include bilateral interstitial infiltrates, numerous centrilobular nodules, and tree-in-bud opacities [1]. On macroscopic examinations of lung tissue, DAB demonstrates miliary or diffusely scattered, yellow tinted nodules. Histologically, the presence of chronic mural inflammation with foreign body reaction within the bronchioles suggests a diagnosis of DAB [2].

## Case presentation

A 55-year-old man with a past medical history of childhood asthma, hyperlipidemia, and GERD presented to the pulmonary clinic for evaluation of chronic cough and dyspnea on exertion. His symptoms began approximately two years prior to presentation and included shortness of breath with persistent coughing and clear sputum production. He was capable of ambulating but his symptoms significantly worsened with activity and walking up stairs. He denied fevers, weight loss, or hemoptysis. 

Additionally, the patient reported a history of severe GERD symptoms associated with significant alcohol use and frequent vomiting/choking on gastric contents, which prompted him to decrease his alcohol intake about one year prior to his presentation. He was a former smoker, having quit 10 years prior to presentation with an estimated total pack-year count of approximately 25 years. The patient denied any other significant substance abuse. At the time of evaluation he owned two dogs and denied ownership of or significant exposure to birds. He previously worked as a saw sharpener for four years during which he was exposed to aerosolized particulate matter but reported wearing an appropriate mask while working. Home medications included atorvastatin, omeprazole, and fluticasone nasal spray. 

**Figure 1:**
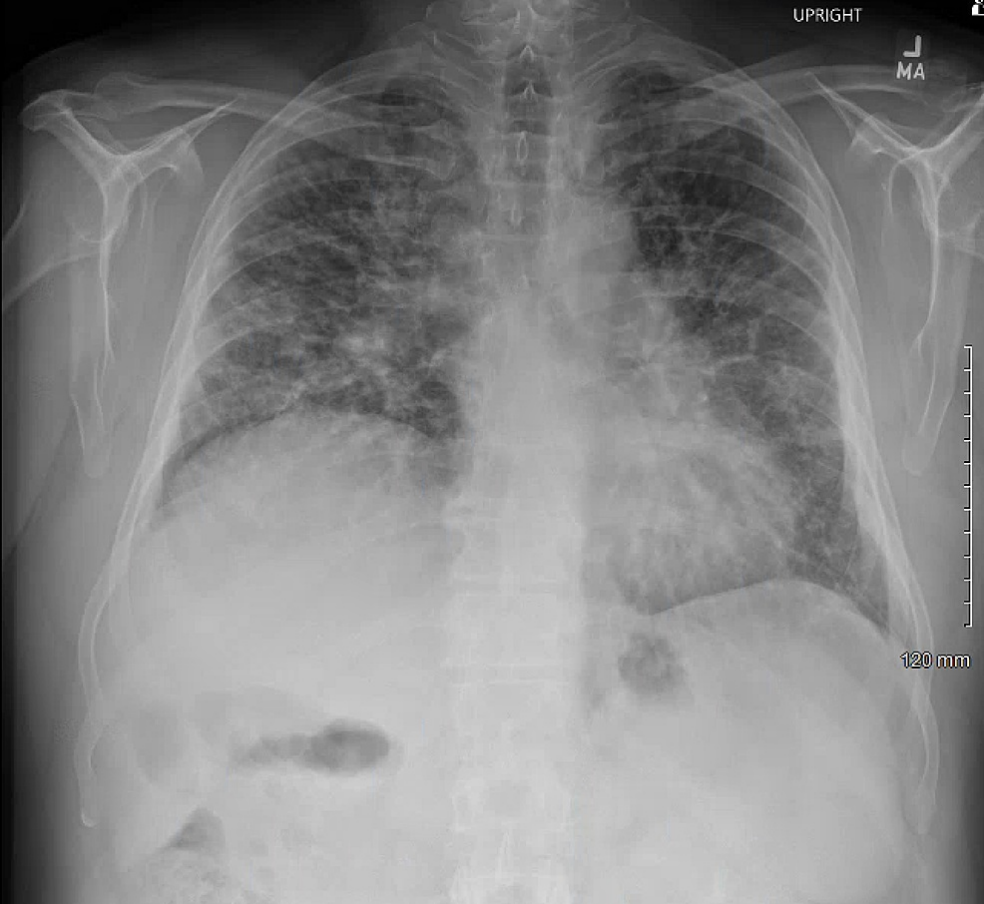
Chest X-ray

**Figure 2:**
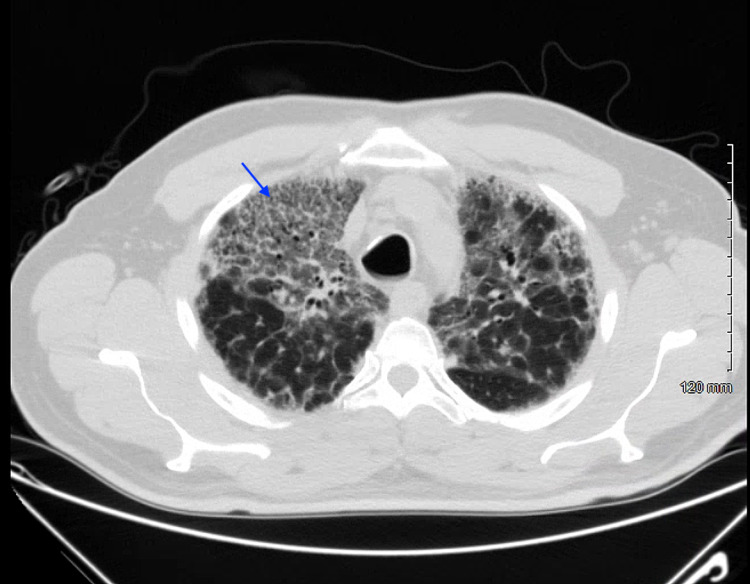
High-resolution computed tomography depicting upper-lobe ground-glass opacities (blue arrow)

**Figure 3:**
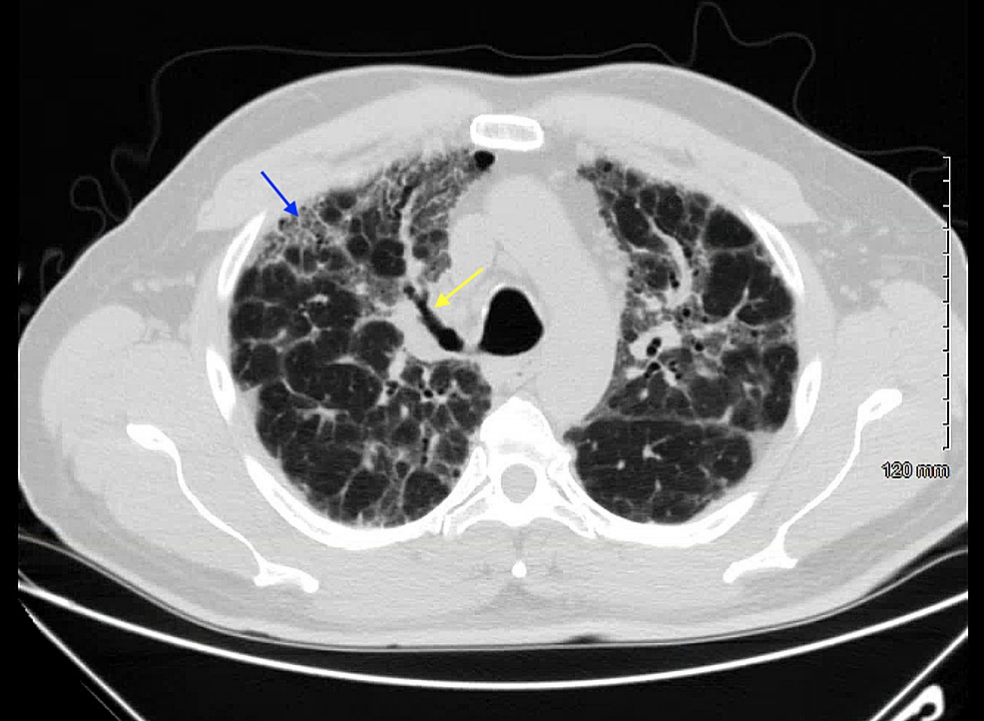
High-resolution computed tomography depicting ground-glass opacities (blue arrow) and bronchiectasis (yellow arrow) at the level of the aortic arch

One month prior to presentation in the pulmonary clinic, he was evaluated in the emergency department where he was diagnosed with pneumonia and prescribed a short course of azithromycin. A chest X-ray was obtained (Figure 1). Subsequently, a high-resolution CT scan of the thorax was obtained with findings of ground-glass opacities bilaterally and bronchiectasis (Figures 2, 3). Further workup for the patient’s lung disease was pursued after evaluation in the pulmonary clinic including basic labs and screening for autoimmune disease which were within normal limits. Pulmonary function testing demonstrated a severe restrictive pattern with total lung capacity of 46% of predicted. A transthoracic echocardiogram was obtained demonstrating an estimated ejection fraction of 55%, mildly enlarged right ventricular cavity with normal function, mild-to-moderate pulmonic regurgitation, and normal-sized right atrium and inferior vena cava. Doppler was suboptimal, preventing estimation of pulmonary artery pressure. Additionally, he was found to be hypoxic and prescribed oxygen via nasal cannula at 2 L/min. 

The patient was then referred for outpatient bronchoscopy and bronchoalveolar lavage. Cell count and differential are presented in Table 1. Culture was positive for *Streptococcus pneumoniae* and scant *Haemophilus influenzae*. Cytology was negative for malignancy. 

**Table 1:** Bronchoalveolar lavage results

Cell Type	Cell Count (%)
Neutrophils	8
Lymphocytes	5
Eosinophils	5
Macrophages	79
Ciliated Epithelial	3

**Figure 4:**
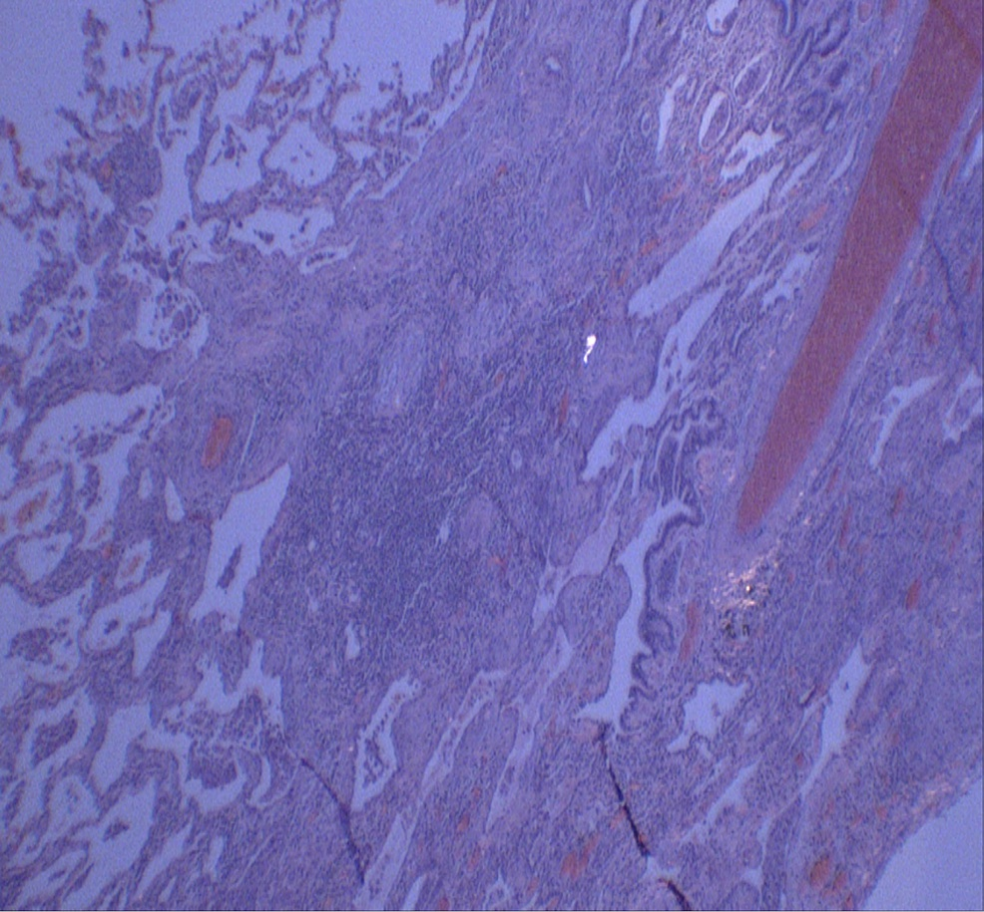
Histopathology demonstrating an airway-centered component and foreign body material

**Figure 5:**
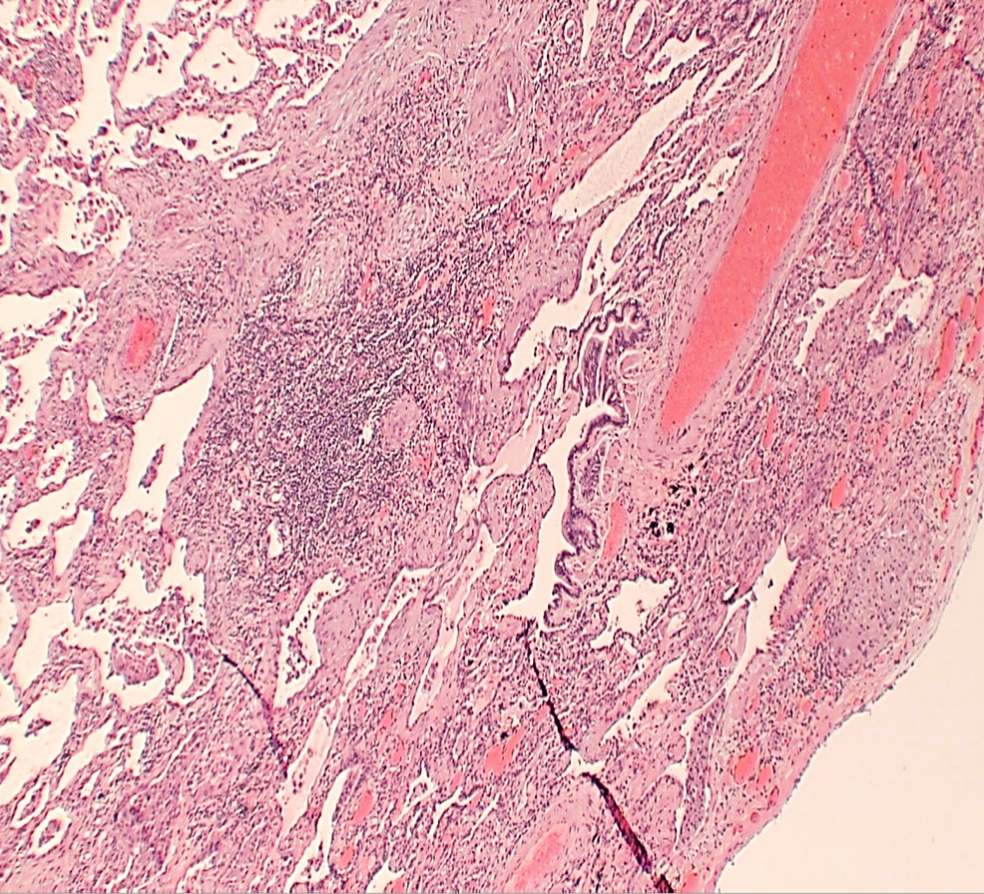
Presence of fibroblastic proliferation and chronic inflammatory cell infiltrate in centrilobular distribution

The patient was then referred to a tertiary care center for evaluation by cardiothoracic surgery. He underwent right middle and lower lobe wedge resection, which found fibrosing interstitial lung disease with an airway-centered component and foreign body material suggestive of chronic aspiration bronchiolitis. Also mentioned in the pathology report was a minor component of usual interstitial pneumonia (UIP) given areas of subpleural fibrosis and fibroblast focus activity (Figures 4, 5).

The patient was re-evaluated in the clinic and encouraged to pursue further workup with gastroenterology for further investigation including swallowing evaluation and pH manometry. At the time of writing, he had not yet completed his gastrointestinal workup.

## Discussion

The patient’s presentation illustrates a complicated case of aspiration-associated lung disease. Radiographically, the patient has predominantly bilateral upper lobe disease, which is consistent with disease from aspiration. The reticular pattern seen on high-resolution CT also suggests aspiration. There is, however, a lack of diffuse bronchiolitis in the form of tree-in-bud opacities that has been associated with a diagnosis of DAB. Additionally, the evidence of lower lobe involvement and subpleural disease combined with the presence of bronchiectasis suggest a UIP pattern in this patient. Histopathology demonstrates similar findings including clear evidence of aspiration disease with the presence of foreign body material.

The exact etiology of this pathology remains debatable and may be related to other exposures in the patient’s history (i.e. working as a saw sharpener) or development of coexisting lung disease that is unrelated to aspiration. However, given the patient’s history of severe GERD symptoms and excessive alcohol consumption associated with recurrent vomiting/choking on gastric contents, we propose that the primary underlying etiology is chronic aspiration. Combining the patient’s history with radiographic and pathologic findings, we favor a diagnosis of chronic aspiration bronchiolitis with features of UIP. 

Clinically, it is important to remember that chronic aspiration often presents subclinically and requires pathologic evaluation prior to diagnosis. In this case, by the time the patient was evaluated for his progressive dyspnea, he was requiring supplemental oxygen therapy and chest imaging was suggestive of advanced disease. Notably, by the time of evaluation, the patient had modified his lifestyle and was no longer consuming alcohol. He also reported adherence to proton-pump inhibitor (PPI) therapy, which had significantly improved his acid reflux symptoms. The possibility of gastrointestinal tract malfunction or an anatomical abnormality can not be excluded as a contributing cause to the patient’s recurrent aspiration. Unfortunately, the patient has not yet followed up with a specialist to undergo swallowing evaluation and endoscopy. 

The treatment of chronic aspiration bronchiolitis is focused on prevention of further aspiration. Under this pretense, if the patient continues to abstain from alcohol and adhere to PPI therapy, his lung disease may stabilize. If, however, the patient has coexisting lung disease from another entity, there may be further progression of his disease. It is worth noting that since the patient’s initial visit to the pulmonary clinic, oxygen therapy with 2 L of nasal cannula has been sufficient to treat his hypoxia and his respiratory symptoms have not worsened.

## Conclusions

Chronic occult aspiration is an important entity that should be suspected by the astute clinician as a cause for chronic lung disease. When evaluating patients with chronic dyspnea, a thorough history should be taken including a detailed account of the patient's social history. If the initial workup including chest radiography and laboratory testing fails to uncover an etiology, a referral to a specialist should be highly considered. Pathology of lung disease from chronic aspiration can be varied as represented by this case, which showcases features of aspiration bronchiolitis with UIP. Treatment of chronic aspiration is focused on correcting the underlying mechanisms of aspiration. This case adds to the small body of documented cases of chronic aspiration bronchiolitis.
